# Geospatial correlates of early marriage and union formation in Ghana

**DOI:** 10.1371/journal.pone.0223296

**Published:** 2019-10-10

**Authors:** Fiifi Amoako Johnson, Mumuni Abu, Chigozie Edson Utazi

**Affiliations:** 1 Department of Population and Health, Faculty of Social Sciences, University of Cape Coast, Cape Coast, Ghana; 2 Regional Institute for Population Studies, University of Ghana, Legon, Ghana; 3 WorldPop and Southampton Statistical Sciences Research Institute (S3RI), University of Southampton, Southampton, England, United Kingdom; Johns Hopkins Bloomberg School of Public Health, UNITED STATES

## Abstract

The practice of early marriage, although acknowledged as a human rights violation, continues to occur in many countries. Different studies have identified the associated factors in many developing countries. However, these factors often assume no geographical variation in these factors within countries. Again, cultural practices and beliefs which strongly influence the acceptance and practices of early marriage vary geographically. In addition, geographic clusters of high rates of early marriage and union formation are also unknown. Thus, area specific correlates of early child marriage are required for the development of location specific policies to aid the eradication of early child marriage. Using data from the 2010 Ghana Population and Housing Census, this study examines the extent of geospatial clustering in early marriage amongst girls and their spatially-varying associated factors at the district level. The findings reveal strong clustering of high early marriage amongst districts in the Upper West, Northern and Volta regions. Nationally, 6.96% (CI = 6.83, 7.08) of girls are married or in union before their 18^th^ birthday. The estimates range from 2.7% in the Jaman North district in Brong Ahafo region to 19.0% in the Gushiegu district in Northern region. Economic factors were observed as important spatially-varying associated factors. The findings suggest that targeted interventions are required in the effort to eradicate the practice in Ghana.

## Introduction

Early marriage and union formation remain a major concern in many low and middle income countries, despite collective efforts by governments, human rights organisations and civil society groups, amongst others to end the practice [1][2]. Although conventions such as the African Charter on the Rights and Welfare of the Child explicitly prohibit child marriage and union formation, contradictions continue to exist between what formal laws prescribe and what traditionally or customarily is the acceptable age of marriage in many societies [3][4]. The customary age of marriage, particularly for girls is much lower than what formal laws prescribe [3][5]. This inconsistency has been exploited in many sub-Saharan Africa settings, resulting in high rates of early marriage in the region [5]. While both boys and girls are married as children, girls are highly disproportionately affected [1]. Statistics show that globally, over 700 million girls were married or entered their first union before their 18^th^ birthday in 2015, with 18% (125 million) of those living in Africa [6]. UNFPA [7] reported that one in three girls in developing countries (excluding China) will be married before they turn 18 years old [8], with those in rural areas being at a higher risk compared to their urban counterparts [7][9].

The 1992 Constitution of Ghana and the children’s Act of 1998 state the legal age of marriage and union formation for both males and females as 18 years and prohibit marriage or union formation before the attainment of this age [7][10]. The Laws of Ghana respect customary and Islamic marriages, but the legal age at marriage is the same for all. Even though, the constitution of Ghana provides freedom of worship for all, the laws of the country are superior to any religious doctrine. It is therefore illegal to marry anyone below 18 years in Ghana, either customarily or religiously. Chapter 1(2) of the 1992 Constitution of the Republic of Ghana, states: “The Constitution shall be the supreme law of Ghana and any other law found to be inconsistent with any provision of this Constitution should, to the extent of this inconsistency, be void” [10]. In this regard, the operational definition adopted for this study refers to all marriages or union formations before 18 years of age.

The 2010 Ghana Population and Housing Census (GPHC) reported that nearly seven percent of children aged between 12 and 17 years have ever married or are in union [11]. Furthermore, the 2014 Ghana Demographic and Health Survey (GDHS) reported that almost one-tenth of women aged 20–49 years were married by age 15 years [12]. At the regional level, the 2011 Ghana Multiple Indicator Cluster Study (MICS) reported a rising trend in early marriage in the northern part of the country as well as the Central, Western and Eastern regions [13]. Estimates from the United Nations Fund for Population Activities indicate that 407, 000 girls, representing one in four girls in Ghana born between 2005 and 2010 married before their 18^th^ birthday [7].

Although, the national level estimates for Ghana may not be as high as in other countries, there are wide regional variations which are alarming [7][11][12][13]. At the local government administrative level, where intervention programmes are implemented, the variations are more likely to be wider, however, much is not known about the levels and disparities. Unequivocally, early marriage and union formation has become a major concern to the Government of Ghana as well as its development partners and civil society on the negative effects to children, in both their formative and adult lives. Often, child brides are married to men much older than themselves and into polygamous marriages [14][15]. They are exposed to systematic physical, psychological and sexual abuse. In addition, they are confronted with social challenges including limited opportunities for education, economic prospects and poor health including exposure to sexually transmitted diseases [5][9][16][17][18][19]. They are also socially isolated from their own family and friends with little or no support to deal with marriage, parenthood, domestic and family duties [7][16][18]. Pregnancy predisposes these young girls to maternal morbidity and mortality, with girls aged 10 to 14 years and those 15 to 19 years estimated to be five and two times, respectively, more likely to die during pregnancy or childbirth when compared to women aged 20 to 24 years [3][7]. The World Health Organisation estimates that infant deaths are 50% higher amongst babies born to mothers aged below 20 years [20].

The effects of early marriage are not only limited to the girls and their households but also undermine the development agenda of countries. Given the effects stated earlier, there is the potential that the Sustainable Development Goals (SDGs) on education, maternal health, poverty, women empowerment and human rights may not be achieved if the problem of early marriage and union formation is not addressed in many low- and middle-income countries.

Global and national statistics are clearly indicative of the problem in many sub-Saharan African countries. However, they mask within-country geographical variations. Given the varying marital cultures and traditions within sub-Saharan Africa, it is probable that the extent of early marriage and union formation will vary substantially amongst local communities with differential and consequential effects. In this study, using data from the 2010 GPHC, we examine at the district level, geospatial clustering of high rates of early marriage and union formation in Ghana and the factors that are spatially associated with the observed clustering. Understanding within country variations, particularly at the district level where programmes are designed and implemented is particularly important for policy guidance, planning, resource allocation and evaluation of programmes aimed at eradicating early marriage and early union formation. Unravelling the geographical variations and their associated spatial factors is important for initiating targeted interventions and strengthening programmes at places where they are most needed. This analysis will also aid the Government of Ghana in the implementation of the recently commissioned National Strategic Framework on Ending Child Marriage in Ghana [21].

## Data

The data for the analysis is derived from 10% of the individual level 2010 GPHC data provided by the Ghana Statistical Service in an anonymised format. The 2010 GPHC is the fifth census conducted in Ghana, since the country attained independence in 1957. The Census Night for the 2010 GPHC was 26th September 2010. The Census enumerated 24,658,823 people, consisting of 12,024,845 males and 12,633,978 females [22]. The enumerated population aged between 12 and 17 years was 3,254,007, comprising 1,640,661 males and 1,613,346 females. The Census reported that 6.95% of girls aged between 12 and 17 years have ever married or are in union. This varies from 5.75% in the Ashanti region to 10.28% in the Northern region, with the Northern, Upper West and Volta regions having the highest estimates [22]. The data for the analysis consist of 157,010 girls aged between 12 and 17 years for whom complete information was available. The district level sample sizes range from 258 to 13,700, with a median of 694.

Following Ghana’s marital laws (The Children’s Act: Act 560) [10][23], the outcome variable for the analysis is binary coded 1 if a girl aged between 12 and 17 years has ever married (married, separated, divorced and widowed) or is in union (informal or living together) and 0 otherwise. Unions are included in the analyses because they are often consummated under customary agreements and girls in such unions perform similar roles as those married under formal laws including childbearing. Surveys such as the DHS and MICS measure early marriage rates based on age at first marriage for women aged 15–49 years. However, in this study, we focused on the current marital status of girls aged 12 to 17 years since the census did not collect data on age at first marriage. Focusing on current age and marital status, we avoid recall bias and the assumption that no one marries before their 15th birthday. The covariates for the analysis were selected based on evidence from the literature [5][17][18][24][25][26], and their availability in the Census. The selected variables, their classification and coding are shown in Table 1. The selected covariates are grouped into demographic, economic and socio-cultural factors to examine their associations with the spatial patterns in early marriage and union formation at the district level. Correlations amongst the variables were examined using the Interval by Interval Pearson's R, Ordinal by Ordinal Spearman Correlation and Nominal by Interval Eta (S1 Table). The results showed very low correlations amongst the variables. Therefore, low potential for multicollinearity.

**Table 1:** Covariate selected for the analysis.

Variables and their classifications	Coding	Type of variable
*Spatial predictors*		
Place of residence	0 = Urban, 1 = Rural	Categorical
*Demographic factors*		
Age in years	0 = 12, 1 = 13, 2 = 14, 3 = 15, 4 = 16, 5 = 17	Categorical
Age of head of household in years		Continuous
Sex of head of household	0 = Male, 1 = Female	
Type of household	0 = Married couple family, female head, 1 = Married couple family, male head, 2 = Non-married couple family, male head, 3 = Non-married couple family, female head, 4 = Non-family, 5 = Female living alone	Categorical
Household size	0 = 1–2, 1 = 3–4, 2 = 5–6, 3 = 7–8, 4 = 9+	Categorical
*Economic factors*		
Educational attainment	0 = No formal education, 1 = Primary, 2 = Junior high, 3 = Senior high or higher	Categorical
Economic activity	0 = Employed, 1 = Unemployed, 2 = Not active	Categorical
Agrarian and non- agrarian households	0 = Agrarian household, 1 = Non- agrarian household	Categorical
Household wealth status	0 = Poorest, 1 = Poor, 2 = Middle, 3 = Rich, 4 = Richest	Categorical
*Socio-cultural factors*		
Religious affiliation	0 = No religion, 1 = Catholic, 2 = Protestants, 3 = Pentecostal/Charismatic, 4 = Other Christian, 5 = Moslem, 6 = Traditionalist, 7 = Other	Categorical
Ethnicity	0 = Akan, 1 = Ga-Dangme, 2 = Ewe, 3 = Guan, 4 = Gurma, 5 = Mole-Dagbani, 6 = Grusi, 7 = Mande, 8 = Other	Categorical
Nationality	0 = Ghanaian, 1 = Other Africans, 2 = Other nationals	Categorical
Disability	0 = No disability, 1 = Single disability, 2 = Multiple disabilities	Categorical

## Methods

Bivariate analyses are conducted to examine the spatial patterns of the percentage of girls who were married or in union at the district level. The global Moran’s I spatial autocorrelation test is then used to examine areas of spatially significant clustering of high early marriages and union formation. Chi-squared test is used to examine the significance of the differences in the percentage of girls who are married or in union by the selected covariates. To examine the extent to which demographic, economic and socio-cultural factors are associated with the observed spatial patterns amongst girls who are married or in union, a Bayesian Geoadditive Semiparametric (BGS) regression technique is adopted [27]. The BGS technique allows for simultaneous estimation of non-linear effects of the continuous covariates as well as fixed effects of the categorical and continuous covariates in addition to unobserved spatial effects (both spatially structured and unstructured).

The outcome variable of interest *y*_*ij*_ is coded 1 if a girl *i* aged between 12 years and 17 years in district *j* is married or in union and 0 otherwise. The outcome variable *y*_*ij*_ thus follows a binomial distribution with expected probability *π*_*ij*_ of being married or in union. The model linking the probabilities *π*_*ij*_ is the logistic model of the form

$$y_{ij}|\eta_{ij}∼B(\pi_{ij})$$ (1)

$$\pi_{ij}=P(y_{ij}=1|\eta_{ij)}=\frac{exp(\eta_{ij})}{1+exp(\eta_{ij})}$$ (2)

where *η*_*ij*_ is the predictor of interest. If we have a vector $x_{ij}^{\prime }={\left(x_{ij1},\ldots ,x_{ijk}\right)}^{\prime }$ of *k* continuous covariates and $\lambda_{ij}^{\prime }={\left(\lambda_{ij1},\ldots \lambda_{ijd}\right)}^{\prime }$ a vector of *d* categorical covariates, then the predictor *η*_*ij*_ can be specified as

$$\eta_{ij}=\alpha \lambda_{ij}^{\prime }+\beta x_{ij}^{\prime }$$ (3)

where *α* is a vector of unknown regression coefficients for the categorical covariates $\lambda_{ij}^{\prime }$, *β* is a vector of unknown regression coefficients for the continuous covariates $x_{ij}^{\prime }$.

To account for non-linear effects of the continuous covariate and the spatial correlation in the proportion of girls who were married or in union before the legal age of marriage, the BGS framework which replaces the strictly linear predictors with flexible semiparametric predictors was adopted. The model is then specified as

$$\eta_{ij}=\alpha \lambda_{ij}^{\prime }+f_{k}x_{ijk}^{\prime }+f^{spat(S_{i})}$$ (4)

where *f*_*k*_(*x*) are non-linear smoothing function of the continuous variable *x*_*ijk*_ and $f^{spat(S_{i})}$ accounts for unobserved spatial heterogeneity at district *j* (*j* = 1,…,*S*), some of which may be spatially structured (correlated) and others unstructured (uncorrelated). The spatially structured effects show the effect of location by assuming that areas which are geographically close are more similar than distant areas, whilst the unstructured spatial effect accounts for spatial randomness in the model. Eq 5 is thus specified as

$$\eta_{ij}=\alpha \lambda_{ij}^{\prime }+f_{k}x_{ijk}^{\prime }+f^{str(S_{i})}+f^{unstr(S_{i})}$$ (5)

where *f*^*str*^ is the structured spatial effects and *f*^*unstr*^ is the unstructured spatial effects and $f^{spat(S_{i})}=f^{str}+f^{unstr}$. In the case of this study, the spatially structured effects depict the extent of clustering of girls who were married or in union and the influence of unaccounted predictor variables that themselves may be spatially clustered ‎or random. The spatially structured effects depict the extent of clustering in the proportion of girls who were married or in union before the legal age of marriage and the influence of unaccounted predictor variables that themselves may be spatially clustered ‎or random. The smooth effects of continuous factors are modelled with P-spline priors, whilst the spatial effects are modelled using Markov random field priors.

The posterior modes of the structured spatial effects and their corresponding probabilities at ‎‎95% nominal level are used to examine spatial correlates of the outcome variable at the district level. The posterior probabilities at the 95% nominal level show districts where early marriage and union formation are statistically significantly high (high positive estimates of the posterior mode), significantly low (high negative estimates of the posterior mode) and where the effects are not significant (estimated posterior mode not significantly different from zero). The estimated posterior mode of the spatial effects characterises unexplained spatially correlated covariate information. Hence, by employing a sequential modelling approach, these were used to identify districts where the demographic, economic and socio-cultural covariates were spatially correlated with early marriage and union formation.

To examine if there exists significant geospatial clustering in the proportion of girls married or in union before the legal age of marriage at the district level, a model containing only the spatial effects was initially fitted (Model 0). Model 1 accounted for the remoteness of the district (predominantly rural versus urbanised districts), whilst Model 2 included the demographic factors. The economic factors are then included in Model 3. In Model 4, the socio-cultural factors are added. Only covariates significant at p<0.05 are retained in the model.

The Akaike weight *w*_*r*_ computed using the Akaike Information Criterion (AIC) is used to examine model suitability. This is the preferred model selection criteria because it accounts for spatial correlation in the selection of variables [28][29]. The Akaike weight *w*_*r*_ for model *r* is expressed as

$$w_{r}=\frac{exp\{-\frac{1}{2}{∆}_{j}(AIC)\}}{\sum_{r=1}^{R}exp\{-\frac{1}{2}{∆}_{r}(AIC)\}}$$ (6)

where Δ_*j*_(AIC) is the difference between the AIC for each model and the model with the lowest AIC and *R* is the number of fitted models. The Akaike weight ranges between 0 and 1, with the sum of all candidate models equal to 1, and analogous to the probability that model *R*_*r*_ is the best model given the available data and all candidate models [30]. The strength of evidence in favour of one model over the other is determined by dividing their Akaike weights. Interpretation of the final results are based on the best fitted model. The statistical software R was used for the analysis [31].

Although Geographically Weighted Regression (GWR) technique provides a spatial structure of the effects (coefficients) of the covariates, the BGS regression approach was preferred because spatial clustering of categorical covariates and potential local collinearity makes GWR results unstable and limits the interpretation of the distributional patterns of the coefficients [32]. Also, GWR limits examination of curvilinear relationships, which could potentially produce false results of non-stationarity [33]. There are also technical challenges with the implementation of binomial functions in GWR packages. The GWR package provides local coefficients but not the corresponding p-values to examine where the covariates are important and where they are not. The GWR functions implemented in the R software are unclear, particularly for binary outcomes and although the ArcGIS software has a user-friendly tool for GWR, they are only available for linear models [34].

## Results

### Univariate analysis

**Fig 1:**
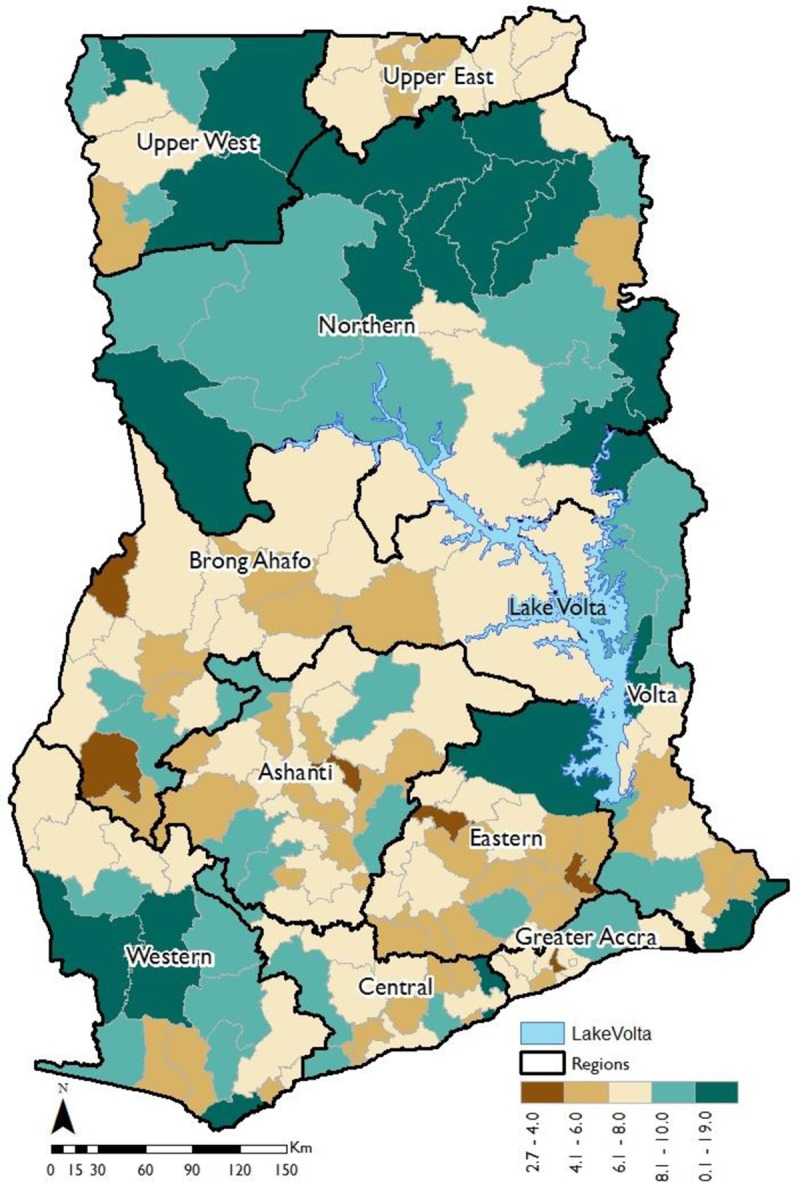
Spatial distribution of early marriage and union formation at the district level.

At the national level, the results of the analysis shows that 6.96% (CI = 6.83%, 7.08%) of girls aged between 12 and 17 years have ever married or are in union. This estimate is similar (6.95%) and within 95% confidence interval of that reported by the Ghana Statistical Service based on the entire Census data [22]. This reinforces the robustness and representativeness of the 10% individual level Census data at the national level. The geospatial variations at the district level in the percentage of girls ever married or in union before the legal age of marriage are shown in Fig 1. The estimates, at the district level range from 2.7% in the Jaman North district in the Brong Ahafo region to 19.0% in the Gushiegu district in the Northern region. Fig 1 shows that early marriages and union formations amongst girls are particularly high amongst districts in the Northern, Upper West and Volta regions. In one-half of all the districts in the Northern region, one in ten girls are married or in union before the legal age of marriage. The global Moran’s I spatial autocorrelation shows a statistically significantly (p<0.01) high positive z-score of 6.27, indicating that early marriage and union formation are not spatially randomly distributed but spatially correlated in Ghana.

Table 2 shows the percentage distribution of girls ever married or in union before the legal age of marriage by the fixed (categorical) covariates. Chi-squared test is used to test for significant differences between the groups. The results show that early marriage and union formation are higher amongst girls in rural communities when compared to their urban counterparts. Considering the demographic covariates, the results show that girls aged between 15 and 17 years are more likely to be married or be in union when compared to their younger counterparts. They are also more likely to be found in non-family type and smaller size households. With regards to the economic covariates, early marriage and union formation are particularly high amongst those with no formal education, unemployed, in agrarian and the poorest households. It is important to note that 14.82% of girls without formal education have married or are in union compared to 5.31% of those with senior high or higher education. Also, more than one in five girls who are not in school and unemployed have ever married or are in union. Analysis of the socio-cultural indicators show that girls with no religious affiliation (12.44%) and those who are traditionalist are more likely to have married or be in union. With regards to ethnicity, the estimates are higher amongst the Gurma and Mole-Dagbani ethnic groups. Considering nationality, girls from other African countries and other nationals are more likely to have ever married or be in union when compared to Ghanaian nationals. Further analysis of the data revealed that amongst the other African countries, the practice is most predominant amongst Burkinabe’s (26.8%), Togolese (9.8%), Nigerians (8.8%) and those classified as other ECOWAS states (7.5%). For other nationals, Asians (7.5%) were more likely to marry or be in union early. The results further show that although the estimates are higher for girls with multiple disabilities, they are not significantly different from those with single or no disability.

**Table 2:** Percentage distribution of girls married or in union before the legal age of marriage by background characteristics.

Background characteristics	% [95% CI]	Sample size	p-value
All	6.96 [6.83, 7.08]	157010	
***Spatial predictors***			
Place of residence			0.000
Urban	5.79 [5.63, 5.95]	82413	
Rural	8.25 [8.05, 8.44]	74597	
***Demographic predictors***			
Age in years			0.000
12	6.13 [5.85, 6.40]	29122	
13	5.59 [5.31, 5.87]	26211	
14	5.67 [5.39, 5.95]	25972	
15	6.64 [6.35, 6.92]	28904	
16	7.15 [6.83, 7.48]	23944	
17	11.23 [10.82, 11.64]	22857	
Sex of head of household			0.000
Male	7.95 [7.78, 8.12]	96305	
Female	5.38 [5.20, 5.56]	60705	
Type of household			0.000
Married couple family, male head	7.87 [7.69, 8.05]	84036	
Married couple family, female head	9.27 [8.54, 10.00]	6019	
Non-married couple family, male head	6.95 [6.49, 7.40]	11821	
Non-married couple family, female head	4.89 [4.71, 5.08]	53831	
Non-family	28.28 [25.4, 31.17]	937	
Female living alone	7.92 [5.15, 10.69]	366	
Household size			0.000
1–2	8.80 [8.06, 9.55]	5600	
3–4	7.27 [6.97, 7.57]	28545	
5–6	6.56 [6.33, 6.78]	45228	
7–8	6.35 [6.09, 6.60]	35301	
9+	7.43 [7.18, 7.68]	42336	
***Economic predictors***			
Educational attainment			0.000
No formal education	14.82 [14.23, 15.41]	13941	
Primary	6.75 [6.55, 6.94]	60680	
Junior high	5.87 [5.70, 6.05]	68810	
Senior high or higher	5.31 [4.93, 5.69]	13579	
Economic activity			0.000
Employed	11.14 [10.77, 11.51]	27466	
Unemployed	21.34 [19.32, 23.36]	1579	
Not active	5.88 [5.75, 6.01]	127965	
Agrarian and non- agrarian households			0.000
Agrarian household	7.56 [7.39, 7.74]	87950	
Non- agrarian household	6.18 [6.00, 6.36]	69060	
Household wealth status			0.000
Poorest	9.03 [8.70, 9.37]	28208	
Poor	7.97 [7.71, 8.23]	42107	
Middle	6.83 [6.50, 7.17]	21799	
Rich	5.94 [5.69, 6.20]	32354	
Richest	4.94 [4.70, 5.17]	32542	
***Socio-cultural predictors***			
Religious affiliation			0.000
No religion	12.44 [11.48, 13.4]	4510	
Catholic	6.10 [5.79, 6.41]	22379	
Protestants	6.04 [5.77, 6.31]	30815	
Pentecostal/Charismatic	6.23 [6.01, 6.45]	47189	
Other Christian	6.75 [6.39, 7.11]	18779	
Moslem	8.40 [8.07, 8.74]	26344	
Traditionalist	10.68 [9.88, 11.47]	5817	
Other	7.82 [6.28, 9.35]	1177	
Ethnicity			0.000
Akan	6.14 [5.97, 6.32]	74113	
Ga-Dangme	6.41 [5.95, 6.88]	10666	
Ewe	7.57 [7.20, 7.93]	20447	
Guan	6.73 [6.09, 7.38]	5776	
Gurma	8.05 [7.49, 8.61]	9054	
Mole-Dagbani	8.20 [7.86, 8.55]	24250	
Grusi	7.83 [6.99, 8.67]	3945	
Mande	6.01 [4.90, 7.12]	1764	
Other			
Nationality			0.003
Ghanaian	6.93 [6.80, 7.05]	153861	
Other Africans	8.56 [7.50, 9.61]	2688	
Other nationals	7.81 [5.36, 10.26]	461	
Disability			0.419
No disability	6.95 [6.82, 7.07]	154355	
Single disability	7.18 [6.07, 8.29]	2075	
Multiple disabilities	8.28 [6.03, 10.52]	580	

CI–Confidence Interval; p-values from Chi-squared test

### Bayesian geoadditive semiparametric regression analysis

**Fig 2:**
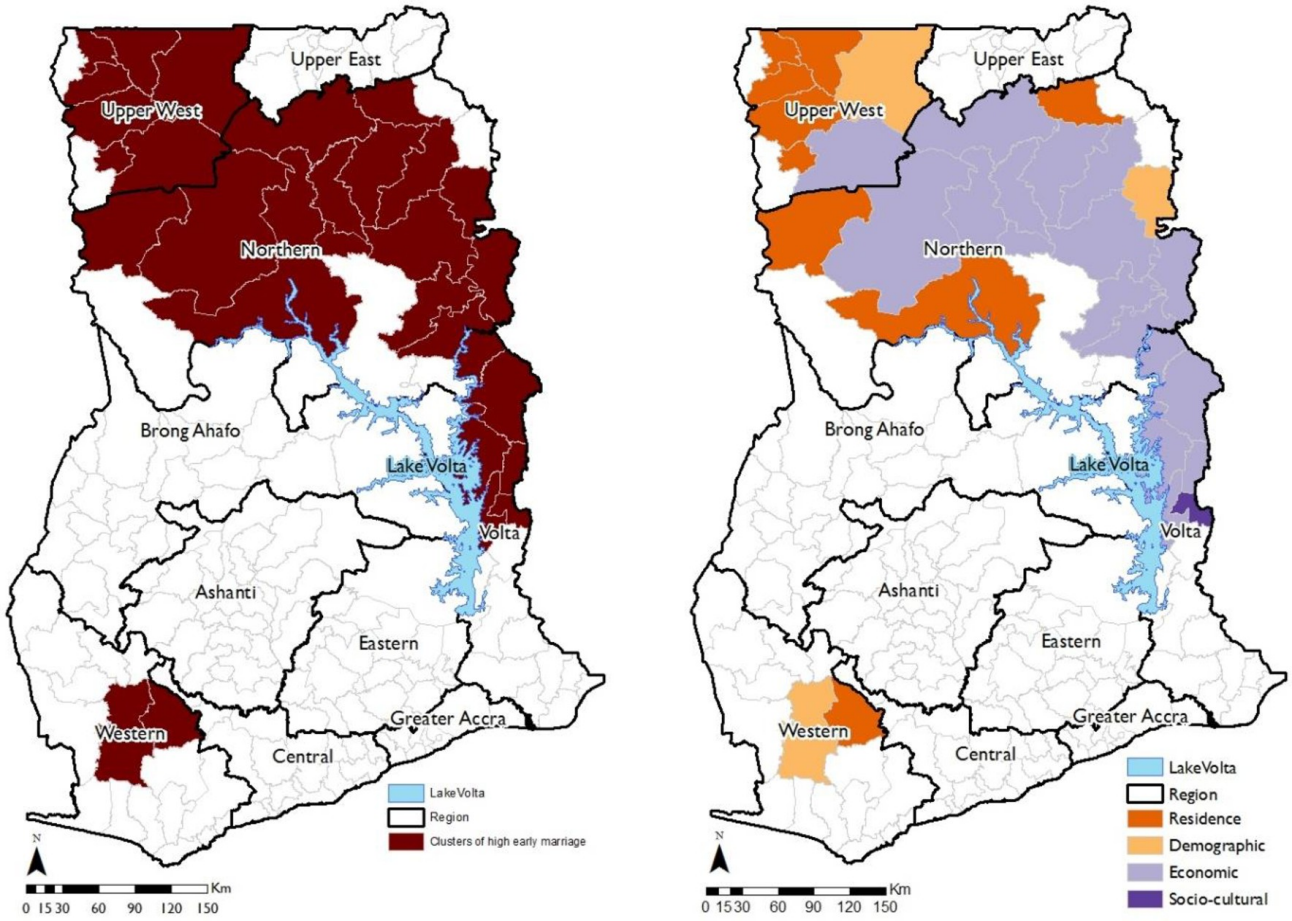
Geospatial (a) clustering of girls married or in union before the legal age of marriage and (b) their associated factors a. clustering of high early marriage and union formation b. correlates of high early marriage and union formation.

The estimated posterior odds ratios of early marriage and union formation amongst girls and their corresponding 95% credible intervals for the fixed covariates are shown in Table 3, along with their model summary statistics. Interpretation of the model coefficients is based on the final model (Model 4). The estimated AIC for Model 0 (null model) is 78,404.4 (Table 3). Fig 2A shows districts where the posterior mode of the structured spatial effects remained positive and significantly high at the 95% nominal level. The figure shows that high early marriage and union formations are concentrated in 30 districts in the Northern, Upper West, Volta and Western Regions.

**Table 3:** Posterior odds of girls married or in union before the legal age of marriage of the fixed effects and their corresponding 95% credible intervals.

Background characteristics	Model 1 POR [95% CI]	Model 2 POR [95% CI]	Model 3 POR [95% CI]	Model 4 POR [95% CI]
***Residential predictor***				
Rural-Urban residence				
Urban	1.00	1.00	1.00	1.00
Rural	1.33 [1.27, 1.40]**	1.34 [1.27, 1.41]**	1.14 [1.08, 1.21]**	1.15 [1.09, 1.21]**
***Demographic predictors***				
Age in years				
12		1.00	1.00	1.00
13		0.95 [0.88, 1.02]	0.97 [0.91, 1.05]	0.98 [0.91, 1.05]
14		0.97 [0.90, 1.04]	1.03 [0.96, 1.11]	1.03 [0.96, 1.11]
15		1.07 [1.00, 1.14]	1.12 [1.04, 1.20]**	1.12 [1.04, 1.20]**
16		1.22 [1.14, 1.31]**	1.31 [1.22, 1.41]**	1.31 [1.22, 1.41]**
17		2.05 [1.92, 2.19]**	2.18 [2.03, 2.34]**	2.17 [2.02, 2.34]**
Sex of head of household				
Male		1.00	1.00	1.00
Female		10.97 [7.53, 15.97]**	12.48 [8.51, 18.30]**	12.22 [8.33, 17.94]**
Type of household/family				
Married couple, female head		1.00	1.00	1.00
Married couple, male head		12.59 [8.55, 18.54]**	14.55 [9.81, 21.57]**	14.17 [9.55, 21.03]**
Non-married couple, male head		0.70 [0.65, 0.76]**	0.72 [0.66, 0.78]**	0.71 [0.66, 0.77]**
Non-married couple, female head		5.83 [3.99, 8.52]**	6.62 [4.50, 9.74]**	6.45 [4.38, 9.49]**
Non-family		6.86 [5.58, 8.45]**	6.07 [4.90, 7.51]**	6.08 [4.91, 7.54]**
Female living alone		2.48 [1.43, 4.31]**	2.74 [1.57, 4.80]**	2.68 [1.53, 4.69]**
Household size				
1–2		1.00	1.00	1.00
3–4		1.09 [0.97, 1.23]	1.11 [0.98, 1.25]	1.11 [0.99, 1.26]
5–6		0.93 [0.82, 1.05]	0.94 [0.83, 1.06]	0.95 [0.84, 1.07]
7–8		0.83 [0.74, 0.95]**	0.82 [0.72, 0.93]**	0.83 [0.73, 0.94]**
9+		0.92 [0.81, 1.04]	0.86 [0.76, 0.98]**	0.87 [0.77, 0.99]*
***Economic predictors***				
Educational attainment				
No formal education			2.54 [2.29, 2.81]**	2.46 [2.22, 2.73]**
Primary			1.59 [1.45, 1.74]**	1.57 [1.43, 1.73]**
Junior high			1.24 [1.14, 1.35]**	1.24 [1.13, 1.35]**
Senior high or higher			1.00	1.00
Economic activity				
Employed			1.00	
Unemployed			2.1 [1.84, 2.41]**	2.06 [1.80, 2.36]**
Not active			0.74 [0.7, 0.78]**	0.74 [0.70, 0.78]**
Household wealth status				
Poorest			1.22 [1.12, 1.32]**	1.19 [1.10, 1.29]**
Poor			1.25 [1.17, 1.35]**	1.24 [1.15, 1.33]**
Middle			1.24 [1.14, 1.33]**	1.22 [1.13, 1.32]**
Rich			1.12 [1.04, 1.20]**	1.10 [1.03, 1.18]**
Richest			1.00	1.00
***Socio-cultural predictors***				
Religious affiliation				
Catholic				1.00
No religion				1.73 [1.55, 1.94]**
Protestants				1.08 [1.00, 1.17]*
Pentecostal/Charismatic				1.04 [0.97, 1.12]
Other Christian				1.11 [1.02, 1.20]*
Moslem				1.22 [1.13, 1.33]**
Traditionalist				1.29 [1.15, 1.44]**
Other				1.26 [1.01, 1.58]*
Ethnicity				
Akan				1.00
Ga-Dangme				1.00 [0.90, 1.10]
Ewe				0.95 [0.88, 1.03]
Guan				1.09 [0.97, 1.24]
Gurma				1.43 [1.28, 1.59]**
Mole-Dagbani				1.17 [1.08, 1.27]**
Grusi				1.13 [0.98, 1.30]
Mande				1.36 [1.10, 1.69]**
Other				0.95 [0.86, 1.05]
Variance components				
Structured Spatial Effect (SSE)	0.5940	0.4149	0.1621	0.1319
% change SSE	+1.3	-30.2	-60.9	-18.6
Unstructured Spatial Effect (USE)	0.051	0.0555	0.0491	0.0502
Model summary statistics				
-2 log-likelihood	‎78,025.6	75,155.2	74,062.9	73,897.6
AIC	78,281.4	75,468.6	74,384.4	74,246.4
Δ_j_(AIC)	4158.0	1222.2	138.0	0.0
Akaike weight	0.00	0.00	0.00	1.00

Model summary statistics for Model 0: -2 log-likelihood = 79,144.5; AIC = 78,404.4, Δ_j_(AIC) = 4158; Variance of SSE = 0.5862; Variance of USE = 0.0581

**Model 0:** Structured and unstructured spatial effects only; **Model 1:** Residential + structured and unstructured spatial effects; **Model 2:** Residential + demographic + structured and unstructured spatial effects; **Model 3:** Residential + demographic + economic + structured and unstructured spatial effects; **Model 4:** Residential + demographic + economic + socio-cultural + structured and unstructured spatial effects.

**p<0.01

*p<0.05

POR–Posterior Odds Ratio; CI–Credible Interval; Δ_j_(AIC) = AIC–AICmin, the difference between the AIC for each model and the model with the lowest AIC

When remoteness (either predominantly rural or urban) of the districts is included in the model (Model 1), the AIC reduced by 123.0. The estimated posterior odds ratios (Table 3, Model 4) show that girls in remote (rural) districts are 15% more likely to marry or be in union when compared to those in urban areas. When the rural-urban covariate is included in the model, the posterior mode of the structured spatial effects become statistically insignificant (p>0.05) in nine of the 30 clustered districts with high rate of early marriage and union formation. These are the Wassa Amenfi East district in the Western region, the Sawla-Tuna-Kalba, Gonja Central and the Mamprusi East districts in the Northern region and the Wa Municipal, Nadowli, Jirapa, Sissala West and Lambussie districts in the Upper West region (Fig 2B). This indicates high early marriage and union formation amongst rural communities in these districts.

**Fig 3:**
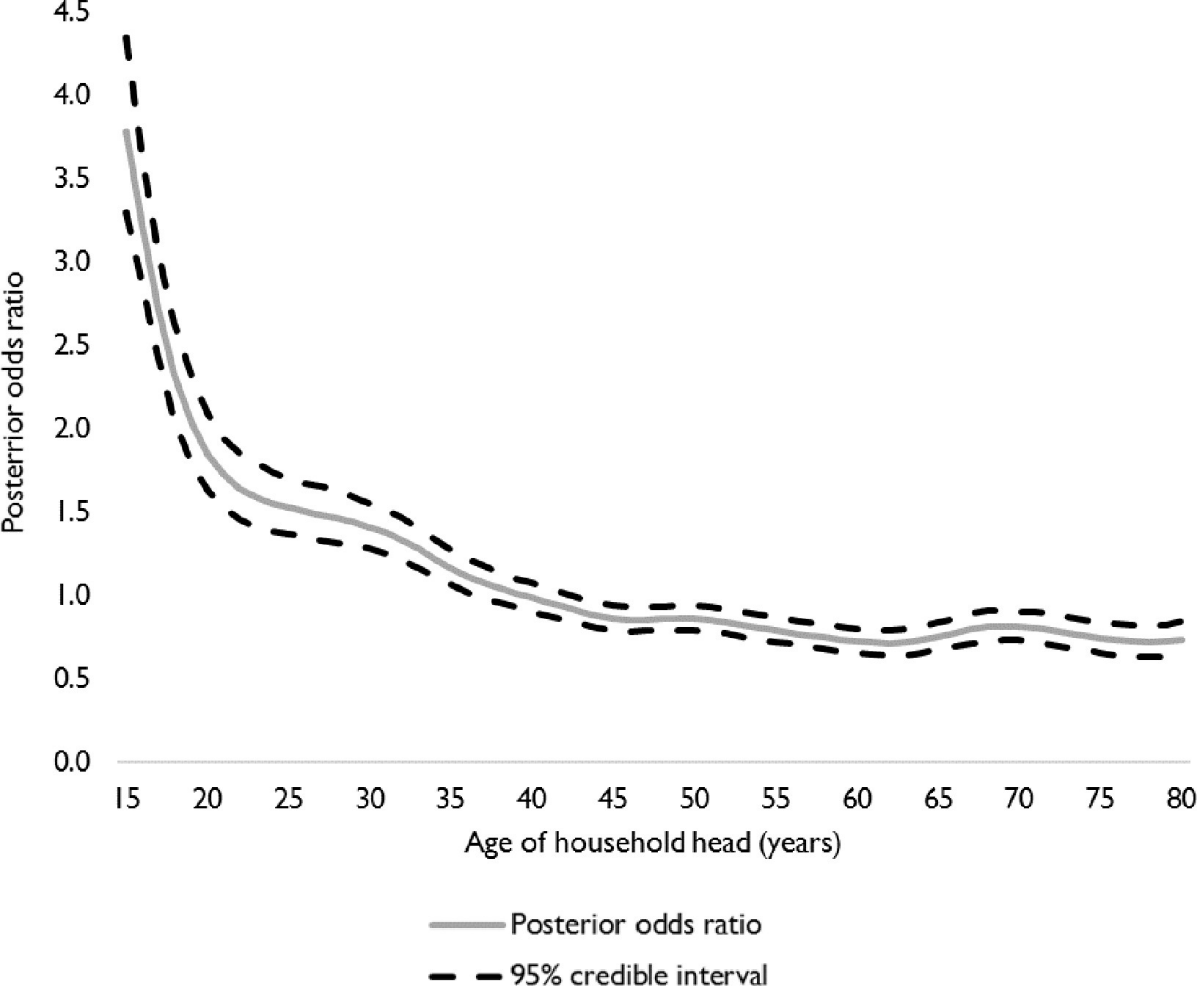
Posterior odds of girls married or in union before the legal age of marriage and their 95% credible interval for the age of household head.

The demographic covariates are included in Model 2, leading to a reduction of 2812.8 in the AIC. The significant demographic covariates are age, sex of the head of household, type of household, household size and age of the head of households (Table 3). The table shows that girls aged 15, 16 and 17 years have significantly higher odds (12%, 31% and 117%, respectively) of marrying or being in union when compared to their 12-year-old counterparts. Early marriage and union formation are also significantly more likely in female headed and smaller households. The posterior odds ratios (Fig 3) show that early marriage and union formation are higher amongst girls in households where the head is aged between 15 and 23 years. The demographic covariates are associated with early marriage and union formation in the Saboba district in the Northern region, the Sissala East district in the Upper West region and the Wassa Amenfi West in the Western region (Fig 2B).

The economic factors are included in model 3, resulting in a decline of 1084.2 in the AIC. The results reveal that educational attainment, economic activity and household wealth status are significantly associated with the odds of girls being in early marriage or union (Table 3). The results show that girls with no formal education, those with primary and junior high school education are 146%, 57% and 24%, respectively more likely to marry or be in union when compared to those with secondary/senior high or higher level education. Girls who are unemployed are twice more likely to marry or be in union compared to those who are employed, while those who are inactive including those in school are 26% less likely to marry or be in union. The economic covariates further show that girls from poor households are significantly more likely to marry or be in union when compared to those from rich households. Fig 2B shows that economic factors are the most predominant predictors, statistically significant in 17 of the 30 (57%) spatially clustered districts with high rates of early marriage and union formation. In the Northern region, the economic covariates are significantly associated with marrying early or union formation in the West Gonja, Nanumba South, Nanumba North, Zabzugu Tatali, Yendi, Tamale Metro, Tolon Kumbugu, Savelugu Nanton, Karaga, Gushiegu and the Mamprusi West districts. They are also significant in the Biakoye, Kadjebi, Krachi East, Nkwanta South and Nkwanta North districts in the Volta region and the Wa East district in the Upper West region.

The AIC reduced by a further 138.0 when the socio-cultural factors are added in Model 4. The results shows that the socio-cultural factors are associated with marrying early or being in a union only in the Jasikan district in the Volta region. The estimated posterior odds ratios (Table 3) show very strong evidence (p<0.01) to suggest that girls with no religious affiliation (73%), Traditionalist (29%), Moslems (22%), Protestants (4%), other Christians (11%) and other religious groups (26%) are more likely to marry early or be in union when compared to Catholics. Having accounted for all the other variables in the model, the estimated posterior odds ratios show that girls from the Gurma, Mole- Dagbani and Mande ethnic groups have increased odds of marrying or being in union before their 18^th^ birthday.

Table 3 shows that when the demographic covariates are included in the model, the estimated variance of the posterior structured spatial effects declined by 30.2%. However, when the economic factors are added to the model, the remaining estimated variance of the posterior structured spatial effects declined by 60.9%. The remaining estimated variance of the posterior structured spatial effects declined by 18.6% when the socio-cultural factors were added to the model. Having accounted for the demographic covariates, the large decline in the estimated variance of the posterior structured spatial effects when the economic covariates were included in the model, coupled with their associative effects in more than half of the spatially clustered districts with high rates of early marriage and union formation, reinforce the importance the economic factors. To ascertain the independent effect of the economic covariates a model with only the economic covariates was fitted. The model showed a similar pattern in the spatial effects as well as reduction in the posterior structured spatial effects when compared with Models 3 and 4 (Table 3).

## Discussion

Using 10% of the individual level data from the 2010 Ghana Population and Housing Census, this study examines hotspots of early marriage and union formation and their spatial correlates (demographic, economic and socio-cultural) at the district level in Ghana. The study makes an important contribution by identifying geographical hotspots of high early marriage and union formation and how demographic, economic and socio-cultural as well as remoteness of geographical locations are spatially associated with early marriage and union formation. The results revealed that 6.96% (CI = 6.83%, 7.08%) of girls aged between 12 and 17 years have ever married or been in union. The findings also show strong clustering of early marriage and union formation amongst districts in the Northern, Upper West and Volta regions. The estimates, at the district level range from 2.7% in the Jaman North district in the Brong Ahafo region to 19.0% in the Gushiegu district in the Northern region, suggesting large geographical variations in the exposure of girls to early marriage and union formation amongst districts in Ghana.

Evidence from our analysis suggests that economic factors (educational attainment, economic activity and household wealth status) are the most predominant factors associated with early marriage and union formation in areas observed to have high early marriage and union formation in Ghana. The economic factors were statistically significantly associated with early marriage and union formation in 17 of the 30 spatially clustered districts with high early marriage and union formation. In those districts, girls with no education, those unemployed and from poor households are significantly more likely to marry or be in union before their 18^th^ birthday. These findings reinforce the social and economic detriments that early marriages and union formation inflicts on the development and wellbeing of young girls, particularly their educational development, participation in the labour market and their opportunities to exiting chronic poverty [35]. Financial insecurities have been a major barrier to eradicating early marriage in many developing societies. Reliance on bride price to support family is a motivational factors for marrying girls off early [36][37][38]. In addition, the younger the bride, the higher the bride price, the higher the value placed on her reproductive capacity, virginity and productive labour [39]. The outcome being low educational achievements for girls, lack of economic opportunities and sexual and reproductive health inequities. Clearly, our findings suggest that girls in the regions of Ghana observed as hotspots of early marriage and union formation rates are not immune from these effects. The 2010 GPHC reported the lowest literacy and educational attainment rates amongst females in the Northern (literacy = 30%, no formal education = 51%) and Upper West (literacy = 40%, no formal education = 43%) regions (GSS 2013).

The study also shows that early marriage and union formation are also more likely (15%) in remote rural districts, which concurs with several studies which examined the determinants of early marriage in sub-Saharan Africa [7][9][40]. Although some studies have established that early marriage is strongly embedded in socio-cultural practices, including ethnicity and religious beliefs [41], in Ghana their effects are trivial amongst hotspots of high early marriage. The socio-cultural predictors were only important in the Jasikan district in the Volta region. The results shows that the Gurma, Mole-Dagbani and Mande ethnic groups, and particularly, Moslems, Traditionalist and those with no religious affiliations are more likely to engage in early marriage and union formation. It is interesting to note that, the Gurma, Mole-Dagbani and Mande ethnic groups, who are predominantly affiliated to the three religions mentioned earlier are one of the major migrant groups in the Jasikan district. The Gurma, Mole-Dagbani and Mande ethnic groups originally migrated from the Northern part of Ghana to engage in subsistence agriculture in the Jasikan district. Culturally, for some of these ethnic groups, menarche symbolises maturity and readiness for marriage. However, scientific evidence shows that age at menarche differ for girls both within and between societies [42]. Some cultures ignore this and operate on the belief that menarche shows readiness for marriage [43]. The consequence being early marriage and union formation [38].

Similar to other studies, the analysis revealed that demographic factors are also strongly associated with early marriage and union formation [18][26][44]. Girls aged 15 to 17 years, those in female headed and large households are the most at risk. Unlike other studies, this analysis specifically identified the Saboba district in the Northern region, the Sissala East in the Upper West region and the Wassa Amenfi West in the Western region as the early marriage and union formation hotspots where the demographic factors are important.

The findings from this study reaffirm the need for targeted interventions in the efforts to eradicate early marriage and union formation. This study, having established the districts where early marriage and union formation are strongly clustered and further identified the spatial associative factors will aid the efforts of the Government of Ghana, particularly in targeting at risk population. Clearly, efforts to eradicate early marriage and union formation amongst girls should not only focus on reforms to the legal and policy framework [21] but also empowering of young girls and their families, provision of information, skills and support networks as well as quality formal education and community social support.

## Supporting information

S1 Table Correlations amongst variables. (XLSX)
